# Prospective Randomised Control Study to Evaluate the Effectiveness of Hypochlorous Acid as a Peritoneal Lavage Agent to Prevent Surgical Site Infection After Exploratory Laparotomy for Perforation Peritonitis

**DOI:** 10.7759/cureus.96059

**Published:** 2025-11-04

**Authors:** Mayank Singh, Arun Kumar, Krishna P Singh, Richa Singh

**Affiliations:** 1 Department of General Surgery, Moti Lal Nehru (MLN) Medical College, Prayagraj, IND; 2 Department of Community Medicine, Moti Lal Nehru (MLN) Medical College, Prayagraj, IND

**Keywords:** hocl, hypochlorous acid, perforation peritonitis, peritoneal lavage, surgical site infection

## Abstract

Introduction

Surgical site infections (SSIs) are a major concern following emergency gastrointestinal procedures, particularly exploratory laparotomy for perforation peritonitis, where infection risk is amplified by the inherent contamination of the operative field. Despite prophylactic antibiotics, the microbial burden frequently surpasses host defence thresholds. Intraoperative peritoneal lavage with antimicrobial agents such as hypochlorous acid (HOCl) has emerged as a promising strategy, combining physical removal of contaminants with direct microbicidal action.

Methods

This was a prospective, randomized controlled trial and 148 patients were included in the study who presented with peritonitis secondary to gastrointestinal perforation. Participants were randomly assigned to receive intraoperative lavage using either HOCl (Group A) or normal saline (Group B). The primary endpoint was the rate of SSIs within 30 days postoperatively. Secondary outcomes included early wound progression, postoperative fever, and rates of wound healing by postoperative days 15 and 30. Additional variables such as patient demographics, comorbidities, surgical parameters, and anatomical factors were evaluated.

Results

The HOCl group demonstrated a marked reduction in SSI incidence (20.3%) compared to the saline group (39.2%), yielding an absolute risk reduction of 19%. Early postoperative indicators - wound severity escalation and fever by day 3 - were predictive of subsequent SSI. No statistically significant associations were found between infection rates and patient-related or operative variables. By day 15, 79% of patients in the HOCl group showed complete wound healing, significantly outperforming the saline group (50%, p<0.001). All HOCl-treated wounds achieved complete healing by day 30. No adverse reactions or resistant organisms were identified among HOCl intraperitoneal lavage recipients.

Conclusion

Intraperitoneal hypochlorous acid lavage has a significant role in reducing the rate of SSI in perforation peritonitis cases.

## Introduction

Surgical-site infections (SSIs) are among the most frequent complications following surgery, affecting approximately 3% of all surgical cases and reaching rates as high as 20% in individuals undergoing emergency intra-abdominal operations [1]. The occurrence of SSIs places a considerable strain on patients by increasing postoperative complications, healthcare costs, and the duration of hospital stays. It also adds a substantial workload for healthcare providers and consumes valuable hospital resources.

According to the Centers for Disease Control and Prevention (CDC), an infection is considered an SSI only if it develops within 30 days after the surgical procedure, or within one year if a medical implant has been placed. SSIs are categorized into three types: superficial incisional infections, deep incisional infections, and organ or space infections.

A superficial SSI is identified when it arises within 30 days following an operation and is confined to the skin or subcutaneous tissue at the site of the incision and one of the following: (a) Purulent discharge, (b) Organisms isolated, (c) At least one sign or symptom of infection, (d) Diagnosis made by a surgeon [2].

Deep incisional SSIs are those that develop within 30 days after an operation and extend into the deeper soft tissues, specifically involving the fascial and muscle layers at the site of the surgical incision. A deep SSI confirmed when it meets at least one of the following conditions: (a) Presence of purulent drainage from the deep layers of the incision, (b) The incision either opens on its own or is intentionally reopened or aspirated by the surgeon, with or without positive culture findings, (c) Detection of an abscess or other clear signs of infection during surgery, on histopathological analysis, or through imaging studies, (d) The patient exhibits at least one clinical sign such as a fever exceeding 38°C, or localized pain or tenderness at the surgical site [2].

The classification of operative wounds is based on the degree of microbial contamination. CDC estimates that, even with antibiotic prophylaxis, the risk of developing an SSI following abdominal surgery ranges from about 2% to 8%, depending on the degree of wound contamination as: Clean- 1-2%, Clean contaminated- 3%, Contaminated- 6%, Dirty- 7% [3].

SSI continues to rank among the leading hospital-acquired complications after emergency gastrointestinal surgery, with reported incidences of 15-45% even in high-volume centres [4]. Exploratory laparotomy for perforation peritonitis is uniquely susceptible because the incision is dirty-infected from the outset; bacterial contamination can exceed 10⁵ CFU/ml before closure and overwhelms host defences despite systemic antibiotics [5]. The concept of intra-operative peritoneal lavage is therefore biologically attractive: by mechanically removing debris, diluting endotoxin, and delivering an antimicrobial fluid directly to the peritoneal cavity, the inoculum may be reduced below the critical threshold required for infection [6].

## Materials and methods

Study design

This was a prospective, randomized, single-blinded, clinically controlled study. This study was conducted over a nine-month period (September 2024-May 2025). This randomized controlled trial was conducted in adherence to the CONSORT 2010 guidelines for reporting randomized clinical trials. The following are the outcomes of the study:

Primary outcome: Incidence of superficial and deep incisional SSIs within 30 days postoperatively, defined according to CDC criteria [2].

Secondary outcomes: Wound healing, postoperative fever, early wound progression, microbiological profile of infected wounds, and any adverse events attributable to HOCl.

Ethical approval and informed consent

The study received approval from the Institutional Ethics Committee (IEC/MLNMC/2024/TP-29) and was prospectively registered in the Clinical Trials Registry of India (CTRI/2024/12/078118).

All patients, or their legally authorized representatives in case of incapacity, provided written informed consent prior to participation. The consent process followed the principles outlined in the New Drugs and Clinical Trials Rules (2019) and the ICH-GCP E6(R3) guidelines. Each participant was provided with a Patient Information Sheet (PIS) and Written Informed Consent Form (WIC) in English and Hindi.

For emergency cases, the following protocol was implemented: When the patient was conscious and hemodynamically stable, informed consent was obtained directly after explaining the purpose, nature, risks, and possible benefits of the intervention in language comprehensible to the patient. If the patient was unconscious or otherwise incapable of providing consent, authorization was obtained from the next of kin or a legally recognized representative. In exceptional life-threatening circumstances where no surrogate was immediately available, the procedure was carried out under the emergency waiver clause, consistent with institutional ethical policies, with proper documentation and subsequent notification to the patient or their representative once the condition stabilized.

*Capacity* *Assessment*

The ability of each participant to provide informed consent was evaluated by the attending surgical resident or consultant before enrolment. Patients were considered competent to consent if they were fully conscious, oriented to time, place, and person, haemodynamically stable, and demonstrated adequate understanding of the study objectives, procedures, potential risks, and benefits, as well as the implications of participation or refusal.

*Consent* *Procedure*

Informed consent was obtained by a member of the surgical research team - either an investigator or a trained resident - well-versed in Good Clinical Practice (GCP) guidelines. The investigator explained the nature and purpose of the study, the operative procedure involved, possible complications, and expected outcomes in language comprehensible to the participant. The discussion was conducted in Hindi or English, according to the participant’s preference, and each individual was provided with a printed copy of the PIS for their review and reference.

The Institutional Ethics Committee reviewed and approved the study protocol, including the consent process and emergency consent provisions, prior to commencement of the research. Blank templates of PIS and WIC, as approved by the Institutional Ethics Committee, are attached as supplementary materials in the Appendix.

Sample size calculation

Sample size was calculated using the formula:

$n = \dfrac{(Z_{\alpha/2} + Z_{\beta})^2 \times \left[ p_1(100 - p_1) + p_2(100 - p_2) \right]}{(p_1 - p_2)^2}$

where,

p1 = Proportion of subjects in which peritoneal lavage with HOCl done

p2 = Proportion of subjects in which peritoneal lavage with NS (0.9%) done

p1-p2 = Clinically significant difference

Zα/2 =1.94

Zβ =0.84

Using the variables from the reference study Singal et al. (2016) in view of wound infection in the above formula,

At a two-sided significance level (1-α) = 95

Power (1-β) = 80%.

$N_{\text(per arm)} = \dfrac{(1.96 + 0.84)^2 \left[0.576(0.424) + 0.384(0.616)\right]}{(0.576 - 0.384)^2}\approx 102.3$

At a finite eligible pool over the recruitment window of nine months at our institution, we applied N≈ 200

n(FPC) per arm = 67.9 (~68 per arm).

Allowing for a 5% attrition rate, the adjusted sample size was calculated 72 per arm.

Randomization and allocation concealment

Randomization was performed using a computer-generated sequence created through an online randomization tool (www.randomizer.org). To ensure allocation concealment, the randomization list was enclosed in sealed, opaque, and sequentially numbered envelopes. Each envelope was opened only after the patient’s enrollment by an independent member who had no role in patient evaluation or data analysis.

Blinding and bias control

Because of the surgical nature of the intervention, blinding of the operating team was not feasible. However, the patients, postoperative care providers, and the independent consultant assessing surgical site infections were kept unaware of the group assignments. Operative notes available to the clinical team were coded and did not reveal the irrigant used. All study data were entered under coded identifiers (Group A and Group B), and the statistician remained blinded to treatment allocation until the final database was unlocked for analysis.

To minimize performance bias, all patients underwent surgery following a standardized operative protocol, with identical surgical techniques, perioperative management, and postoperative care, as outlined in the institutional guidelines. To limit detection bias, outcome evaluations - including postoperative infection rates, wound healing, and recovery parameters - were conducted by independent assessors who were unaware of the treatment allocation and had no participation in the intraoperative procedures.

Study setting and participants

This study was carried out by the Department of Surgery, M. L. N. Medical College at S.R.N. Hospital, Prayagraj, for a period of nine months from September 2024 to May 2025. Those patients were included in this study who underwent exploratory laparotomy for perforation peritonitis, emergency surgery by midline or transverse laparotomy for perforation peritonitis, age ≥ 18 years, American Society of Anesthesiologists (ASA) score ≤ 3 [7], ability to understand the nature and extent of the study, and who had given written informed consent. Those patients were excluded who were unbale to give/understand informed consent, evidence of leak following primary repair of the perforation site/from other sites, pregnancy, laparoscopic surgery done for perforation peritonitis, multiple trauma or organ injury besides perforation peritonitis, revision surgery (previous abdominal surgery within the last 30 days), patients on long term immunosuppressive drugs/history of chemotherapy in last six months, previous history of chronic kidney disease requiring dialysis (eGFR <60 ml/min), previous history of liver disease (Child Pugh score class B and class C) [8], patients allergic to HOCl, concurrent abdominal wall infections, patients lost to follow up, expired, absconded, who withdrew consent before completion of required follow-ups of 30 days.

Data collection included detailed history and clinical examination, full blood count, blood biochemistry, liver function test (LFT), operative findings including perforation site, post-operative wound infection rate and culture and sensitivity pattern of wound discharge.

Study procedure

**Figure 1:**
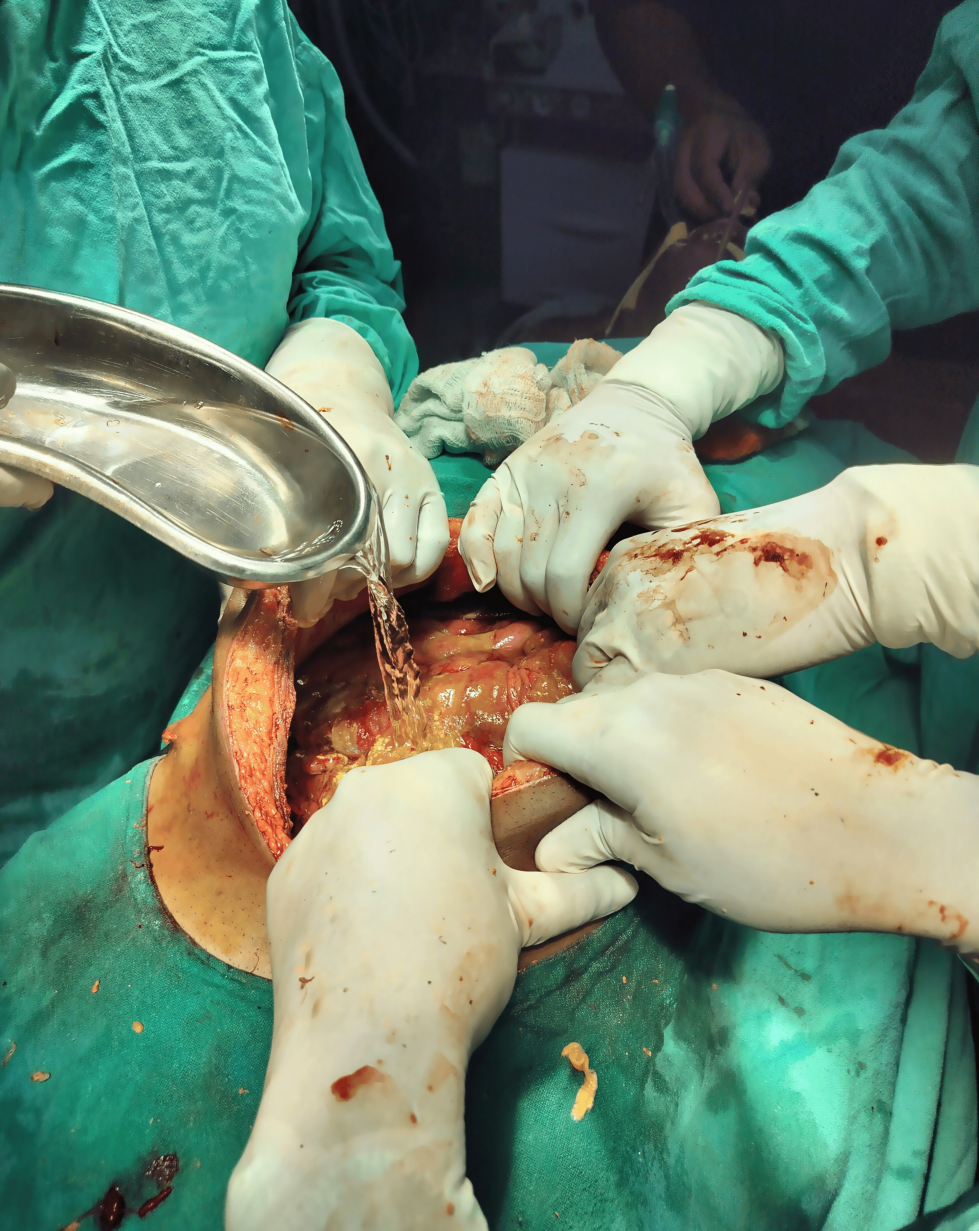
Intraoperative peritoneal lavage with 50 ppm HOCl solution. HOCl: Hypochlorous acid

**Figure 2:**
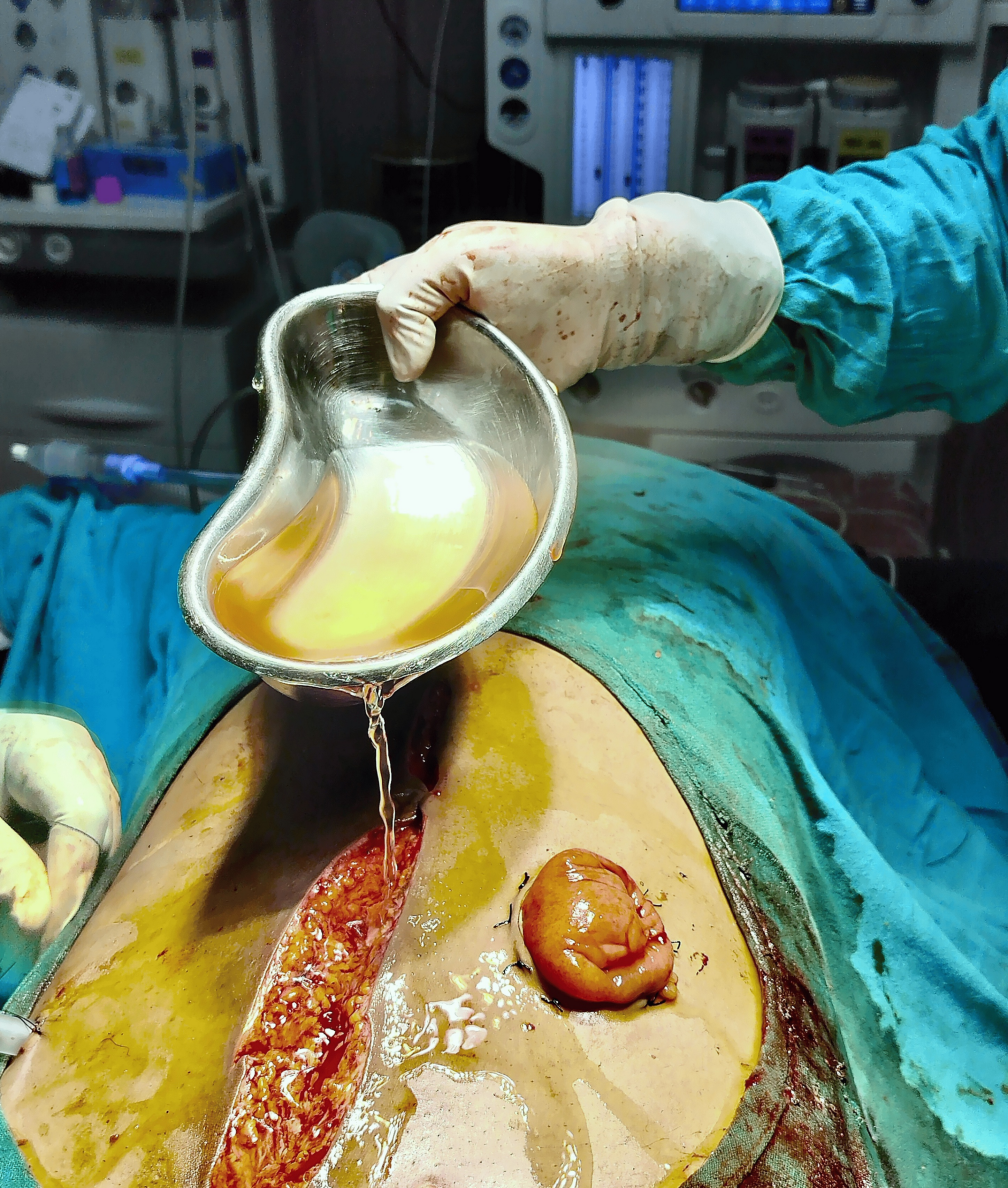
Irrigation with 500 ml of 50 ppm HOCl solution after rectus sheath closure. HOCl: Hypochlorous acid

All patients admitted to the emergency surgery department needing laparotomy for perforation peritonitis were potential candidates for the study. Pre-operative necessary investigations were done. Intravenous antibiotics (III generation cephalosporins - ceftriaxone 50 mg/kg + metronidazole i.v. as per body weight) were given to all patients at induction of anaesthesia. At laparotomy, intra-op findings were noted. A decision was taken with regard to the operative procedure to be performed, and patients were included in the study if they met the inclusion criteria. Randomization was performed using computer-generated random numbers from www.randomizer.org [9]. Group A was the intervention group and Group B was the control group. All individuals admitted in the intervention group (Group A) undergoing laparotomy had intraperitoneal lavage with 2000 ml HOCI (50 ppm) (Figure 1) during exploratory laparotomy, 100 ml of HOCl was left in the abdominal cavity and drain was clamped for 1 hour and closure of the rectus sheath followed by irrigation of the subcutaneous space with 500 ml HOCI (50 ppm) (Figure 2). All individuals undergoing laparotomy in the control group (Group B) received intraperitoneal lavage with 2000 ml normal saline (0.9%), closure of the rectus sheath followed by irrigation of the subcutaneous space with 500 ml normal saline (0.9%).

Post-operatively, patients were closely monitored on POD 3, 7, 15, and 30 for signs of SSI. In patients with infected wounds, discharge was sent for culture sensitivity, and findings were noted. Standard practice of wound care, i.e., regular moist dressing, was done, and antibiotics were guided by the culture and sensitivity results from the infected wounds.

Outcome assessment and follow-up

To maintain consistency and objectivity in postoperative outcome assessment, all evaluators received structured training prior to the start of the study. The training emphasized the use of the Southampton Wound Assessment Scale (SWAS) for grading surgical site wounds, as this validated tool provides an objective framework for classifying wound healing and infection severity. Before commencing data collection, assessors demonstrated satisfactory inter-observer reliability, achieving a Cohen’s kappa value greater than 0.8 on pilot case evaluations.

Patients were monitored at postoperative intervals of 3, 7, 15, and 30 days. Individuals unable to attend in-person follow-up visits were contacted by telephone to document wound status and confirm recovery progress. Those who could not be reached despite repeated efforts (at least three phone calls and one registered letter) were designated as “lost to follow-up.” These cases were excluded from the per-protocol analysis but retained in the intention-to-treat analysis for evaluation of the primary outcomes.

Safety and monitoring adverse events

All patients were closely monitored intra- and post-operatively for any local or systemic adverse effects related to the use of hypochlorous acid (HOCl) lavage. Parameters included wound appearance, local irritation, delayed healing, unexpected inflammation, and systemic reactions (fever, electrolyte imbalance, allergic manifestations).

A dedicated safety monitoring team, independent of the surgical operators and data analysts, reviewed each case daily until discharge and again during scheduled follow-up visits at 3, 7, 15, and 30 days. Any adverse events were documented and graded according to the Common Terminology Criteria for Adverse Events (CTCAE v5.0). Serious adverse events were immediately reported to the Institutional Ethics Committee as per institutional policy.

Statistical analysis

All statistical analyses adhered to the intention-to-treat (ITT) principle, meaning that every randomized participant was evaluated within the group originally assigned, irrespective of protocol deviations or completion of follow-up. This methodology maintains the advantages of randomization and minimizes bias resulting from post-randomization exclusions. To control for possible confounders - such as age, existing comorbid conditions, and the anatomical site of perforation - multivariate analyses were conducted. Binary logistic regression was applied for categorical variables, while analysis of covariance (ANCOVA) was used for continuous variables, with adjustments made for baseline characteristics showing significant intergroup differences (p < 0.10).

Missing data were limited. When encountered, they were addressed using multiple imputation, assuming the data were missing at random (MAR). Sensitivity analyses were subsequently performed to verify the stability and reliability of the findings.

The statistical analysis was performed with SPSS (Statistical Package for the Social Sciences) version 21.0 (IBM Corp., Armonk, NY, USA). The data were presented in the form of mean (standard deviation) and percentage (%). The chi-square test was used to compare categorical variables, while the independent t-test was used to assess discrete variables between groups. Pearson correlation was used to assess the strength and direction of the linear relationship between two continuous variables. A p-value of <0.05 was considered statistically significant.

## Results

**Figure 3:**
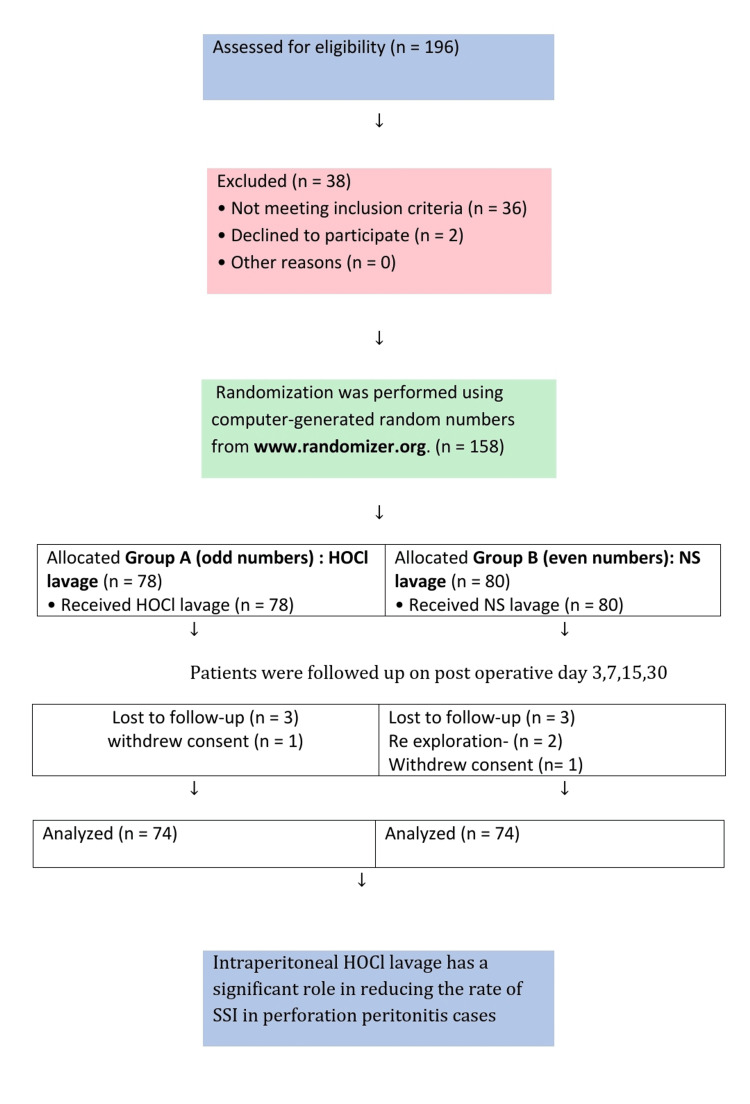
CONSORT Flow Diagram. CONSORT: Consolidated Standards of Reporting Trials; SSI: Surgical site infection Randomization was done from www.randomizer.org [9].

During the period of the study, 196 patients were assessed for eligibility, and 38 were excluded. The study involved a total of 158 patients, where Group A had 78 individuals and Group B had 80 individuals, as shown in the Consolidated Standards of Reporting Trials (CONSORT) flow diagram (Figure 3). During the study period, six patients were lost to follow-ups, two patients withdrew consent, and two patients who needed re-exploration were excluded. Analysis was done for 148 patients, and the results are presented here.

The majority of participants fall within the 41-50 years age range, making up 25% of the overall sample. This is closely followed by the 21-30 years group, which represents 23.6% of the total population. Among those receiving HOCl (Group A), the 21-30 years group forms the largest segment (31.1%). In contrast, participants in the NS group (Group B) were particularly 41-50 years (29.7%) and 31-40 years (28.4%).

The <20 years and 71-80 years categories show the lowest participation, contributing only 2.7% and 4.1% to the total, respectively. Patients aged 51-60 years are fairly evenly split between the two groups - 21.6% in Group A and 14.9% in Group B (Table 1).

**Table 1:** Age distribution.

Age group (years)	Group A, HOCl (%)	Group B, NS (%)	Total, (%)
<20	03 (4.1)	1 (1.4)	4 (2.7)
21-30	23 (31.1)	12 (16.2)	35 (23.6)
31-40	07 (9.5)	21 (28.4)	28 (18.9)
41-50	15 (20.3)	22 (29.7)	37 (25.0)
51-60	16 (21.6)	11 (14.9)	27 (18.2)
61-70	5 (6.8)	6 (8.1)	11 (7.4)
71-80	5 (6.8)	1 (1.4)	6 (4.1)

Out of the total 148 patients, 92 (62.2%) were males and 56 (37.8%) were females. The male-to-female ratio in the study group was 1.6:1. Group A shows 52.7% were males and 47.3% were females. In Group B, 71.6% of participants were male and 28.4% were female. The most frequently observed site of perforation across both groups was the gastric region, accounting for 30.4% of all cases (n=45), followed by ileal perforation in 27.0% (n=40) and duodenal perforation in 24.3% (n=36). Anatomical location of gastrointestinal perforation correlates with the development of SSIs among 148 patients. Of these, 44 patients developed SSIs, while 104 did not (Table 2).

**Table 2:** Site of perforation vs SSI distribution. SSI: Surgical-site infection

Site of Perforation	Absent (n=104)	Present (n=44)	Total (n=148)
Appendix	9 (8.7%)	5 (11.4%)	14 (9.5%)
Ascending Colon	1 (1.0%)	1 (2.3%)	2 (1.4%)
Caecum	0 (0.0%)	1 (2.3%)	1 (0.7%)
Duodenum	24 (23.1%)	12 (27.3%)	36 (24.3%)
Gastric	33 (31.7%)	12 (27.3%)	45 (30.4%)
Ileum	28 (26.9%)	12 (27.3%)	40 (27.0%)
Jejunum	7 (6.7%)	1 (2.3%)	8 (5.4%)
Rectum	1 (1.0%)	0 (0.0%)	1 (0.7%)
Transverse Colon	1 (1.0%)	0 (0.0%)	1 (0.7%)

The most frequently involved site was the gastric region, accounting for 45 cases (30.4%). Among them, 33 patients (31.7%) remained free from infection, whereas 12 (27.3%) developed SSIs. Ileal perforations were also commonly observed, contributing to 40 cases (27.0%), with 28 (26.9%) in the non-infected group and 12 (27.3%) in the infected group. Duodenal perforations were present in 36 patients (24.3%), and of these, 24 (23.1%) had no SSI, while 12 (27.3%) developed infections.

Appendicular perforation was reported in 14 cases (9.5%), with nine (8.7%) showing no infection and five (11.4%) developing SSIs. Less commonly involved sites included the jejunum (5.4%), ascending colon (1.4%), cecum (0.7%), rectum (0.7%), and transverse colon (0.7%). These contributed minimally to the overall burden and showed no strong trend toward infection development.

The Chi-square analysis revealed a P-value of 0.554, indicating no statistically significant association between the perforation site and the occurrence of SSIs in this cohort. In summary, the anatomical site of gastrointestinal perforation does not appear to significantly influence the risk of developing postoperative infection.

A total of 148 patients were evaluated for the presence of surgical site infections (SSIs). In Group A (HOCl group), SSI was absent in 59 patients (79.7%) and present in 15 patients (20.3%). In contrast, Group B (normal saline group) had a higher incidence of SSI, with 29 patients (39.2%) developing infection and only 45 (60.8%) remaining free of it. When both groups were combined, the overall rate of SSI was 29.7% (44 out of 148 patients). Statistical analysis revealed a significant difference between the two groups (p = 0.012), indicating that the use of HOCl was associated with a significantly lower incidence of surgical site infections compared to normal saline. More SSIs occur in males (31/92, 34%) than in females (13/56, 23%), but the difference was not statistically significant (p = 0.176) (Table 3).

**Table 3:** Number of patients with SSI. SSI: Surgical-site infection

SSI	Group A (HOCI)	Group B (NS)	Total (%)	P-value
Absent	59 (79.7)	45 (60.8)	104 (70.3)	0.012
Present	15 (20.3)	29 (39.2)	44 (29.7)	

In the present study, Staphylococcus aureus was the most frequent isolate (34.1%), followed by E. Coli (25%). Other pathogens included S. epidermidis (11.4%), Methicillin-resistant Staphylococcus aureus (MRSA) (9.1%), Klebsiella and Pseudomonas (each 4.5%). No growth was seen in 11.4% of samples. Notably, MRSA was absent in Group A (HOCl) but present in 13.8% of Group B (NS) cases, indicating a possible advantage of HOCl in limiting resistant infections (Table 4).

**Table 4:** Dominant bacteria isolated from infected wounds of patients. SSI: Surgical site infection; MRSA: Methicillin-resistant Staphylococcus aureus

Micro-organism	SSI in Group A (HOCI)	SSI in Group B (NS)	Total (n=44)
S. Aureus	7 (46.7)	8 (27.6)	15 (34.1)
E. Coli	3 (20)	8 (27.6)	11 (25)
Klebsiella	0 (0)	2 (6.9)	2 (4.5)
Pseudomonas	1 (6.7)	1 (3.5)	2 (4.5)
MRSA	0 (0)	4 (13.8)	4 (9.1)
S. epidermidis	2 (13.3)	3 (10.4)	5 (11.4)
No growth	2 (13.3)	3 (10.4)	5 (11.4)

Fever on postoperative day (POD) 3 is markedly associated with later SSI. Almost half of those who developed SSI were febrile on POD 3 (46%), versus 27% when infection was absent. Correspondingly, apyrexia predominated among non-infected patients (73%) but fell to 55% in the SSI group. The chi-square test (p = 0.028) confirms this statistically significant relationship, highlighting early fever as a useful infection warning signal. No statistically significant association was found between the incidence of SSIs and patient factors (age, sex), patient-related indices (BMI, HbA1c), perioperative (incision length, operative time), and anatomical factors (site of perforation, stoma formation).

## Discussion

The present prospective, randomized controlled study evaluated HOCl lavage versus standard normal saline lavage during emergency laparotomy for perforation peritonitis. Our primary outcome demonstrated a significant absolute risk reduction of 19% (20.3% vs 39.2%) in overall SSI at 30 days. The following discussion contextualises each variable relative to the current literature, highlights areas of concordance and discordance, and outlines the limitations and future trajectory of HOCl research.

The 50% relative reduction in SSI observed in Group A is closely aligned with PLaSSo Randomized Clinical Trial (Sellappan et al., 2024) [10]. The incidence of surgical site infection was 15.6% in the super-oxidised solution group compared with 37.2% in the saline group, corresponding to a relative risk of 0.42, and the significant reduction reported in the Kubota et al. study (0% vs 20%) [11]. In contrast, Mueller et al. found no benefit from polyhexanide irrigation during elective laparotomy [12], suggesting that the antimicrobial spectrum, dwell-time, and tissue compatibility differ between agents. The super-oxidised solution evaluated by Singh et al. in perforation peritonitis reduced the incidence of SSI from 40% to 26.67%, an effect size identical to ours [13]. Collectively, these data reinforce the unique potency of HOCl against polymicrobial peritoneal contamination and support its inclusion in future perioperative bundles.

We observed a bimodal age pattern but no age-related SSI risk (p = 0.682). Several large observational cohorts identify advanced age (>65 years) as an independent predictor of SSI owing to immunosenescence and comorbidity [14]. Conversely, Akter et al. noted similar infection rates across decades in 90 patients undergoing emergency laparotomy for perforation, and this mirrored our findings [15]. The discrepancy may reflect the overwhelming influence of contamination in class IV wounds, which can eclipse modest immunologic decrements associated with ageing. Importantly, HOCl efficacy was consistent across all age strata, underlining its suitability even in geriatric cohorts, a point not addressed in earlier trials. Although males predominated overall, gender did not significantly influence SSI in our multivariate analysis (34% vs 23%, p = 0.176). Some registries attribute slightly higher SSI rates to males, possibly due to androgen-modulated immunity or behavioural factors [16]; however, others find no sex effect after adjustment for wound class and obesity [17]. Our neutral result aligns with the CDC‘s recent report that sex was not a determinant of the standardised infection ratio (SIR) across procedure categories [18]. The incidence of SSI reduction seen in both males and females supports the broad application of HOCl.

Gastric, ileal and duodenal perforations comprised >80% of cases. Neither anatomical site nor presence of faeculent contamination predicted SSI [14]. Nevertheless, HOCl efficacy did not vary by perforation site, echoing its broad bactericidal coverage against both Gram-negative enterics (E. Coli) and Gram-positive cocci (Staphylococcus) detected in our culture profile. Pathogenic growth was restricted to SSI-positive wounds. Infection was dominated by Staphylococcus aureus (34%) and E. Coli (25%). These organisms reflect endogenous skin and enteric flora and align with global SSI surveillance data [18]. HOCl oxidative mechanism disrupts bacterial membranes, DNA, and viral capsids within 15s [19], explaining the absence of growth in HOCl-treated patients despite identical antibiotic prophylaxis. Notably, no resistant MRSA phenotype emerged, suggesting that HOCl irrigation may help curb antimicrobial resistance, a benefit highlighted by WHO [20]. The effect of traditional risk factors - BMI >30 kg/m², surgery >180 min - was absent in our cohort (mean BMI 22.7 kg/m², mean duration 2.2 h). Their lack of variance may explain why they did not influence SSI, in contrast with the National Healthcare Safety Network data, where a 2% SIR rise correlated with longer operative times [17]. These neutral variables nevertheless reinforce the internal validity of randomisation, eliminating confounding between groups.

Limitations and future scope

Several caveats warrant discussion. First, the study was single-centre and powered for a 20% absolute difference; rare events such as deep organ-space SSI or mortality were too infrequent to analyse. Second, blinding of operating surgeons was impossible, raising the potential for unconscious bias in wound handling; however, outcome assessors were masked to allocation, and objective CDC criteria were used. Third, the irrigation protocol used a fixed 2.6 L volume of 0.005% HOCl; whether larger volumes or higher concentrations yield incremental benefit remains unknown. Fourth, microbial culture was limited to aerobic bacteria; anaerobes and fungi, though less common, were not assessed. Fifth, follow-up ended at 30 days; late incisional hernias or adhesive complications were outside the study scope. Future research should therefore pursue multicentre, double-blinded RCTs comparing HOCl with other evidence-based irrigants such as povidone-iodine or polyhexanide, stratified by wound class and operative duration. Dose-response studies could identify the minimal effective concentration, facilitating cost-effectiveness analyses - an essential step for resource-limited settings. The antimicrobial-resistance footprint of HOCl deserves genomic exploration, given preliminary data suggesting reduced selection pressure versus antibiotics. Additionally, point-of-care fluorescence imaging could quantify real-time bacterial burden pre- and post-lavage, providing mechanistic insight into irrigation efficacy. Finally, incorporation of patient-reported outcome measures (PROMs) such as pain, cosmetic satisfaction, and quality of life will ensure that future trials capture dimensions valued by patients and payers alike.

## Conclusions

Based on the findings, this study clearly concludes that HOCl significantly reduces the incidence of both superficial and deep incisional SSIs. Patients treated with HOCl lavage showed a 19% absolute and nearly 50% relative risk reduction in overall SSI rates when compared to normal saline lavage. Additionally, early wound assessment indicators such as POD3 pyrexia emerged as reliable predictors of impending infection.

## Figures and Tables

**Figure 4:**
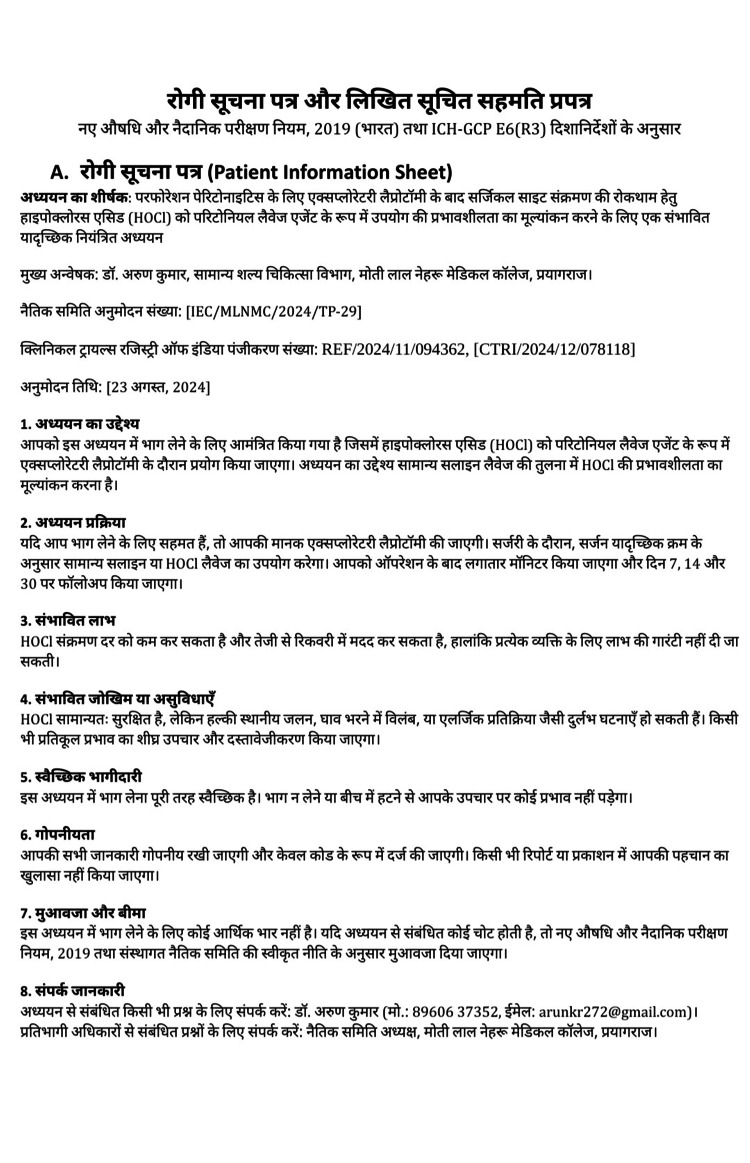
Blank Patient Information Sheet (Hindi) as approved by Institutional Ethics Committee.

**Figure 5:**
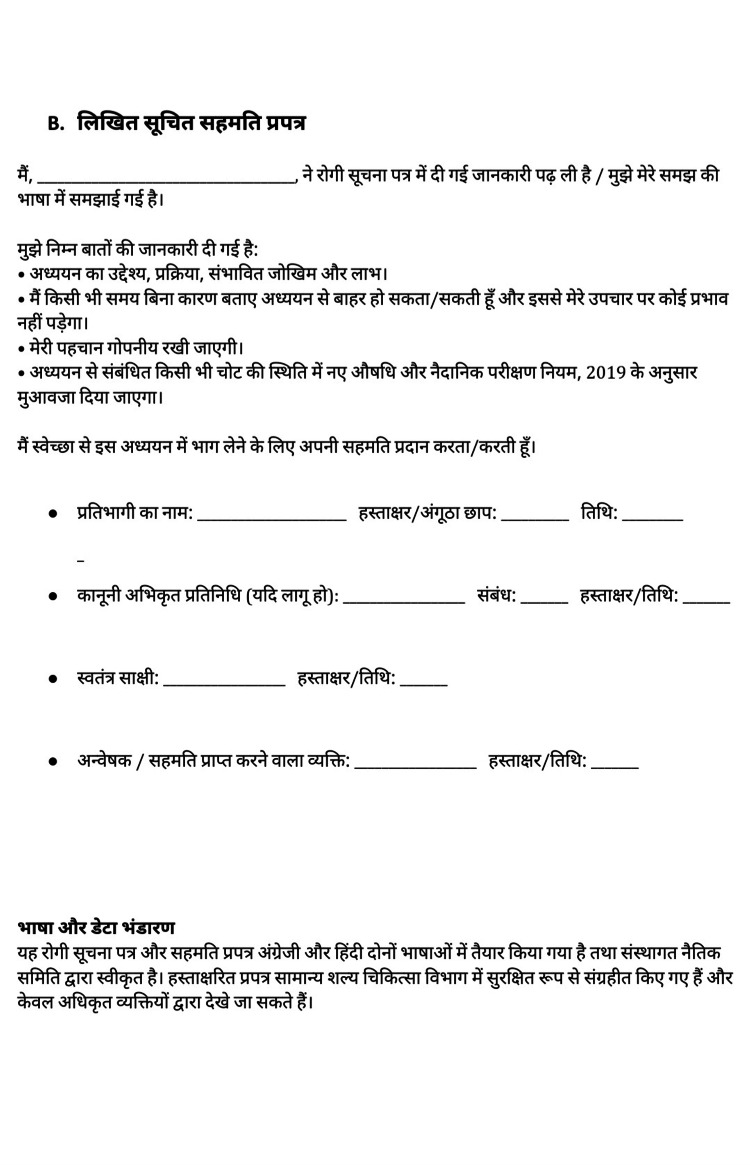
Written Informed Consent (Hindi) as approved by Institutional Ethics Committee.

**Figure 6:**
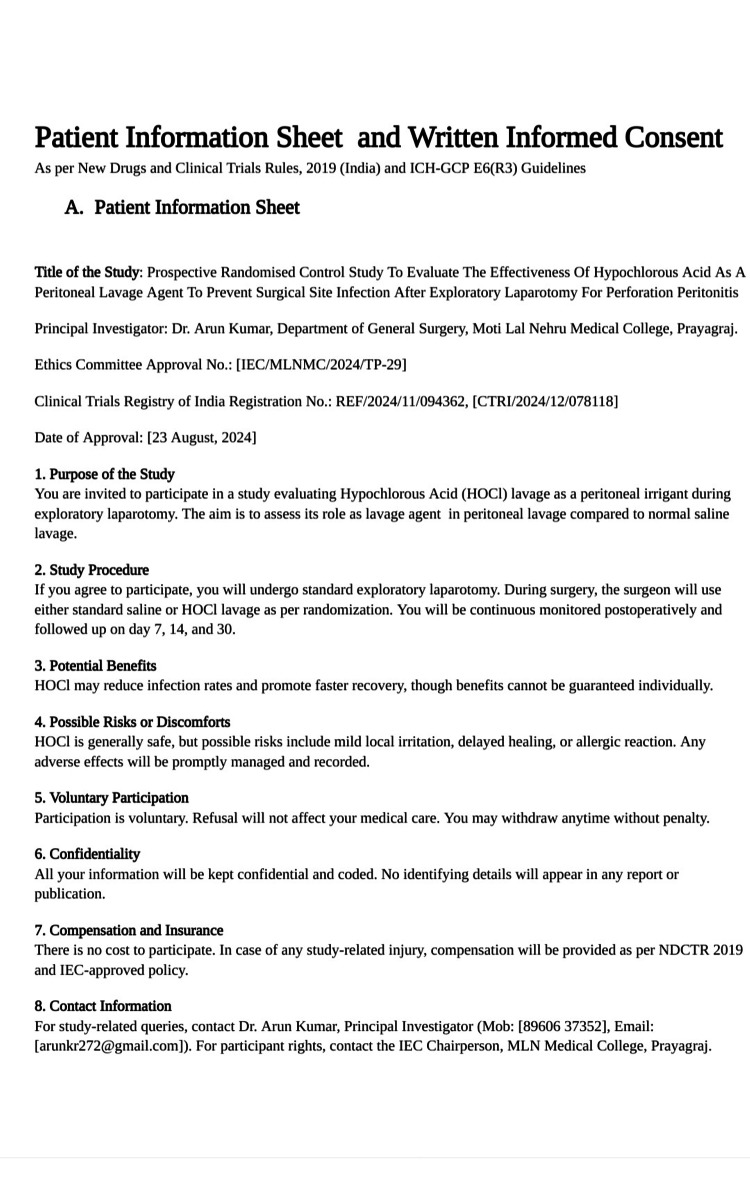
Blank Patient Information sheet (English) as approved by Institutional Ethics Committee.

**Figure 7:**
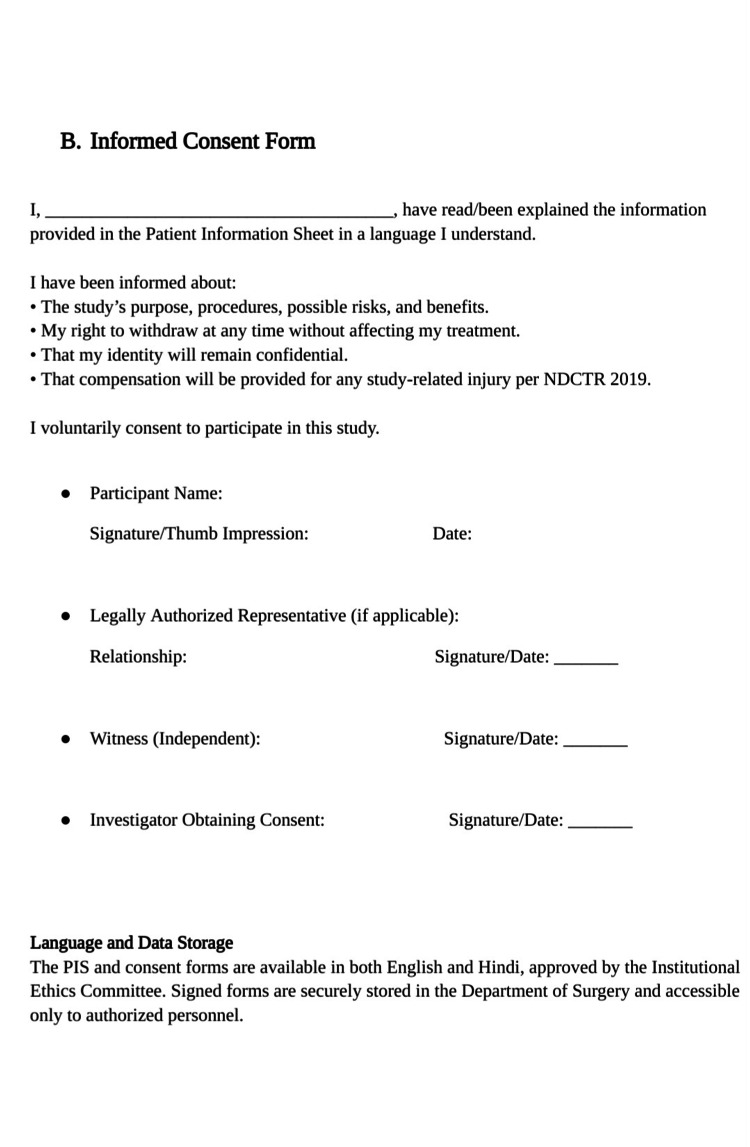
Blank Written Informed Consent form (English) approved by Institutional Ethical Committee.
